# Gasdermin D Plays an Oncogenic Role in Glioma and Correlates to an Immunosuppressive Microenvironment

**DOI:** 10.3390/biom13060904

**Published:** 2023-05-29

**Authors:** Mengyuan Li, Ping Jiang, Shuhua Wei, Yuhan Yang, Liting Xiong, Junjie Wang, Chunxiao Li

**Affiliations:** Department of Radiation Oncology, Peking University Third Hospital, Beijing 100191, China; limengyuan@bjmu.edu.cn (M.L.);

**Keywords:** glioma, GSDMD, prognosis, tumor microenvironment, immune cell infiltration

## Abstract

Background: Understanding the molecular mechanisms driving oncogenic processes in glioma is important in order to develop efficient treatments. Recent studies have proposed gasdermin D (GSDMD) as a newly discovered pyroptosis executive protein associated with tumorigenesis. However, the precise effect of GSDMD on glioma progression remains unknown. Methods: The expression levels of GSDMD in 931 glioma and 1157 normal control tissues were collected. A series of bioinformatic approaches and in vivo and in vitro experiments were used to investigate the roles and mechanisms of GDSMD in glioma. Results: Significant upregulation of GSDMD was detected in glioma tissues compared to normal brain tissues. Patients with glioma and higher GSDMD levels had shorter overall survival, and the Cox regression analysis revealed that GSDMD was an independent risk factor. In addition, upregulation of GSDMD was associated with higher tumor mutation burden and PD-1/PD-L1 expression. Immune infiltration and single-cell analyses indicated that GSDMD was positively associated with an immunosuppressive microenvironment with more infiltrated macrophages and cancer-associated fibroblasts. Furthermore, the in vitro and in vivo experiments revealed that GSDMD knockdown inhibited glioma proliferation, migration, and growth in vivo. Conclusion: Our analyses revealed a relatively comprehensive understanding of the oncogenic role of GSDMD in glioma. GSDMD is a promising prognostic biomarker and a potential target for glioma treatment.

## 1. Introduction

Glioma is the most common malignant tumor of the central nervous system with high invasiveness and lethality, accounting for approximately 70% of intracranial tumors [1]. Based on the degrees of cellular malignancy, glioma is also categorized into four grades (I, II, III, and IV) by the World Health Organization (WHO) [2], while glioma with grades I-III is also termed low-grade glioma (LGG). Despite the advancement of comprehensive treatment, such as surgery, chemotherapy, and radiotherapy, the prognosis of patients with glioma remains poor [3]. For example, glioblastoma multiform (GBM) is the most frequent and highly malignant intracranial tumor, and patients with GBM regularly have a median survival of fewer than 15 months [4]. In addition, cytological and gene expression variety makes it more difficult to develop precision treatments. Thus, it is imperative to explore underlying molecular mechanisms and find novel biomarkers for prognosis and target therapy.

Gasdermin D (GSDMD), a member of the gasdermin family, has been identified as a key factor in the genesis of pyroptosis [5] and secretion of several inflammatory mediators, such as IL-18 and IL-1β [6]. The inflammatory factors released during the process of pyroptosis will lead to the development of inflammation [7,8]. Over the past few years, the effects of molecules, inflammasomes, gasdermins, and inflammatory products during pyroptosis on tumorigenesis have been well investigated, but the conclusions are controversial [9,10]. Previous studies have found that targeting GSDMD can effectively prevent pyroptosis and inflammation and suppress tumorigenesis in lung cancer [10,11]. However, the roles of GSDMD in glioma genesis and progression have not been elucidated.

In recent years, advances in multicolor flow cytometry and single-cell sequencing technology have made it possible to analyze tumor microenvironmental components [12,13]. Researchers have noted that the tumor microenvironment (TME) is an inherent component of the tumor and plays a key role in tumor progression [14]. Some immune cells, such as CD8+ T cells, dendritic cells (DCs), and natural killer (NK) cells, mainly exert anti-tumor effects [15]. In addition, the anti-tumor effects can be restrained by some immunosuppressive cells, such as cancer-associated fibroblasts (CAFs), M2-type macrophages, regulatory T cells (Tregs), and myeloid-derived suppressor cells (MDSCs), which can induce a chronic inflammatory microenvironment and promote tumor proliferation and angiogenesis [16,17,18]. Moreover, the components of TME immune cells are in dynamic states and can be regulated. For example, an inhibitor to immune checkpoint PD-1 can induce an increase in anti-tumor immune cells to suppress tumor growth, which has succeeded in treating glioma clinically [19]. Therefore, exploring the characteristics of the TME of glioma and factors that influence the dynamic changes of the immune microenvironment contributes to a better understanding of glioma and finding novel targets to improve the patient’s prognosis.

Herein, we not only identified GSDMD as a prognostic biomarker for patients with glioma but also revealed the significant effects of GSDMD on remodeling the tumor immune microenvironment. Furthermore, we performed in vivo and in vitro experiments that revealed knocking down GSDMD is a novel target in glioma treatment.

## 2. Methods

### 2.1. Patient Cohorts and Data Acquisition

A total of 931 glioma patients (LGG and GBM) with RNA-sequencing expression profiles, corresponding clinical and follow-up information from the TCGA (https://portal.gdc.cancer.gov/, accessed on 5 March 2022) and GEO (https://www.ncbi.nlm.nih.gov/geo/, accessed on 5 March 2022) databases, and 1157 normal brain tissues from the GTEx (https://gtexportal.org/, accessed on 5 March 2022) database were included in this study. A single-cell expression matrix with 33,685 genes on 20,278 cells was also downloaded from the GEO database (GSE138794). The cohort of 667 glioma patients, including 508 LGG and 159 GBM, from TCGA was used for differential expression analysis of GSDMD and almost all subsequent mechanistic analyses, because the data in this cohort are more comprehensive. Gene expression profiles were downloaded from the UCSC (http://genome.ucsc.edu, accessed on 5 March 2022) database. The fragments per kilobase million (FPKM) values of gene expression were used for all gene-related analyses. The GEO cohort (GSE16011) was used for the validation of expressed differences of GSDMD in normal and glioma tissues and its capacity to predict prognosis.

### 2.2. Cell Culture and siRNA Transfection

The human glioma cell lines U87 and A172 were obtained from the Cell Bank of the Chinese Academy of Sciences (Shanghai, China). The cells were cultured in DMEM (MACGENE, Beijing, China) culture medium supplemented with 1% penicillin/streptomycin and 10% fetal bovine serum (FBS). GSDMD siRNAs (Sequences: si-1, S: 5′-GCCAUCUGAGCCAGAAGAATT-3′; AS: 5′-UUCUUCUGGCUCAGAUGGCTT-3′. si-2, S: 5′-GACACAGAAGGAGGUGGAATT-3′; AS: 5′-UUCCACCUCCUUCUGUGUCTT-3′. NC, S: 5′-UUCUCCGAACGUGUCACGUTT-3′; AS: 5′-ACGUGACACGUUCGGAGAATT-3′.) were purchased from GenePharma company (Hangzhou, China) and the transfection assays were performed with lipo2000 (Invitrogen, Carlsbad, CA, USA) according to their protocol. All cells were maintained at 37 °C with 5% CO_2_.

### 2.3. qRT-PCR

A PrimeScript™ RT reagent Kit (Perfect Real Time) (TAKARA, RR037A) was used for mRNA cDNA synthesis. Subsequently, the expression levels of GSDMD were measured using qRT-PCR with SYBR II Premix Taq (Abclone). 2 µL of cDNA was used as the template for each PCR reaction. GAPDH was used as the internal control. The fold-expression levels of GSDMD were calculated using the 2^−ΔΔCT^ method. All experiments were carried out in triplicate. The primer sequences were as follows: F: GTGTGTCAACCTGTCTATCAAGG; R: CATGGCATCGTAGAAGTGGAAG.

### 2.4. Western Blot

Equal amounts of protein were separated using 12% SDS-PAGE and then transferred to PVDF membranes (Millipore, Billerica, MA, USA). The membranes were blocked using 5% non-fat milk in Tris-buffered saline with Tween-20 (TBS-T). The blots were incubated with GSDMD (1:1000, Proteintech, Rosemont, IL, USA) and actin (1:3000, Proteintech, Rosemont, IL, USA) antibodies overnight at 4 °C. After washing the membranes with TBS-T 3 times (10 min/wash), we incubated them with IRDye 800CW goat anti-mouse (LI-COR Biosciences, #926-32210, Lincoln, NE, USA) and donkey anti-rabbit (LI-COR Biosciences, #926-32213) secondary antibodies at 37 °C for 1 h. We repeated the washing step with TBS-T and the protein signals were analyzed using an Odyssey CLx imager (LICOR Odyssey, Lincoln, NE, USA).

### 2.5. CCK-8 and Colony Formation Assay

U87 cells were seeded into a 96-well plate at a density of 2000 cells/well. After adding 10 µL Cell Counting Kit-8 (CCK-8) (Dojindo, Kumamoto, Japan) into each well at 4, 24, 48, and 72 h, the absorbance values (OD) of transfected cells at 450 nm were measured using a microplate reader (Thermo Fisher Scientific, Waltham, MA, USA). For colony formation assay, U87 cells were seeded into a 24-well plate at a density of 1000 cells/well. The number of cell colonies was counted after 7 days and analyzed using ImageJ software (version 2.0.0). Each experiment was performed in triplicate.

### 2.6. Transwell Assay

Transwell chambers with an 8-µmol/L pore size (Corning, Toledo, OH, USA) were used to detect the invasive capability of the glioma cells. After transfection for 24 h, approximately 5 × 103 transfected U87 cells were transferred into the upper chamber and cultured with serum-free medium, while F12K medium (HyClone) with 20% FBS was added into the bottom chamber. Subsequently, the cells were cultured for 24 h. Then, 4% paraformaldehyde was used to fix the invasive cells, and thereby 0.5% crystal violet dye solution was utilized to stain-fix these cells. Invasive cell numbers were photographed and counted using an optical microscope.

### 2.7. Animal Experiments

Female nude mice (5–6 weeks) purchased from Peking University Health Science Center were raised under specific pathogen-free (SPF) conditions. The Animal Care and Use Committee of Peking University Third Hospital approved all animal experimentation before implementation. For subcutaneous transplantation, 1 × 10^7^ U87 cells were subcutaneously injected into the back of the right forelimb. Subsequently, the body weight and tumor volume were measured at two-day intervals. Seven days after transplantation, the tumor nodules were palpable. The tumor volume was calculated as follows: length × width^2^ × 0.5. When the tumor volume exceeded 1000 mm^3^, the mice were considered death [20]. After 21 days following transplantation, the mice were sacrificed by cervical dislocation and tumor samples were collected.

### 2.8. Immunohistochemistry (IHC)

To evaluate the expression levels of GSDMD protein, IHC results of GSDMD protein expression in glial cells of normal cerebral cortex tissues and glioma cells were obtained from the Human Protein Atlas (HPA, http://www.proteinatlas.org/, accessed on 6 March 2022) database and analyzed.

### 2.9. Pathway Correlation Analysis

To analyze the effects of GSDMD on glioma development and progression, we used the R software (version 4.1.0) GSVA package to evaluate the correlation between GSDMD levels and pathway scores of tumor proliferation and angiogenesis and tumor mutation burden (TMB). The parameter of the method was set to ‘ssgsea’.

### 2.10. Survival Analysis

The Overall Survival (OS) and GSDMD expression levels of LGG and GBM patients were downloaded from the TCGA database (https://portal.gdc.cancer.gov/, accessed on 5 March 2022). The median GSDMD expression was used as the cut-off value to divide the samples into GSDMD low-expression (GSDMD_low) and GSDMD high-expression (GSDMD_high) groups. Survival curves were plotted using the Kaplan–Meier method, and differences were evaluated using the log-rank test. The Cox regression analysis was used for evaluating mortality risks.

### 2.11. Genetic Alteration Analysis

Among these included patients, genetic alteration data of 655 glioma patients are available. The data on the mutation type, alteration frequency, copy number alteration (CNA), and mutated site information of the glioma cells were collected. The chi-square test was used to analyze the difference in genetic alterations between the GSDMD_low and GSDMD_high groups. OS was compared for glioma with or without genetic alterations.

### 2.12. GO/KEGG Enrichment Analyses and Gene Set Enrichment Analysis (GSEA)

The differentially expressed genes (DEGs) between GSDMD_low and GSDMD_high patients were performed using the R package limma. The Gene Ontology (GO)-based enrichment analysis of DEGs was achieved by the topGO R packages to better understand the gene category and function. In addition, The Kyoto Encyclopedia of Genes and Genomes (KEGG) [21] is a database for determining the path of the gene cluster and correlated functions (http://www.genome.jp/kegg/, accessed on 5 March 2022). The KEGG enrichment analysis was performed using the Kobas database (http://kobas.cbi.pku.edu.cn/genelist/, accessed on 5 March 2022) [22] to identify signaling pathways related to the DEGs in KEGG pathways.

To further evaluate the differences in enriched gene sets between the GSDMD_low and GSDMD_high groups, GSEA [23] was carried out using GSEA software (version 4.1.0) (http://software.broadinstitute.org/gsea/index.jsp, accessed on 5 March 2022).

### 2.13. Immune Infiltration Analysis and Single-cell Analysis

The correlation between GSDMD expression and immune infiltration in glioma was analyzed using R package IOBP. Based on the global gene expression of patients, infiltrating scores of B cells, CD4+ T cells, CD8+ T cells, macrophages, natural killer (NK) cells, endothelial cells, cancer-associated fibroblasts (CAFs), and other cells in each tumor were calculated using the ‘deconvo_epic’ method.

The cell types of each sample and GSDMD expression in different glioma cells were analyzed by the 10X genomics single-cell and analysis on 10 glioma (GBM) samples. Related information and data were downloaded from the GEO database (GSE138794). All samples were untreated human gliomas. The cells were profiled using 10X Genomics and mapped to human genome reference sequence version 38 (hg38), R package Seurat was used for the analysis PCA, and UMAP analyses were performed using the “RunPCA” and “RunUMAP” methods. After calculating the proportion of each cell type for each sample, the association between different cell types was analyzed using the Pearson method. Cellular communication was analyzed using the “CellPhoneDB” function.

### 2.14. Statistical Analysis

Statistical analyses and figure plots were completed using the SPSS Statistics 25 (IBM Corp., Armonk, NY, USA), R software (version 4.1.0, http://www.r-project.org, accessed on 5 March 2022) and GraphPad Prism 8.0 (GraphPad Software Inc, San Diego, CA, USA) software. The Mann–Whitney U test package was used to identify DEGs. Chi-square tests were performed to analyze the categorical variables. The Cox regression analysis was carried out by SPSS software (version 25). All statistical tests were two-sided, and statistical significance was set at * *p* < 0.05.

## 3. Results

### 3.1. GSDMD Is Highly Expressed in Glioma Tissues

**Figure 1:**
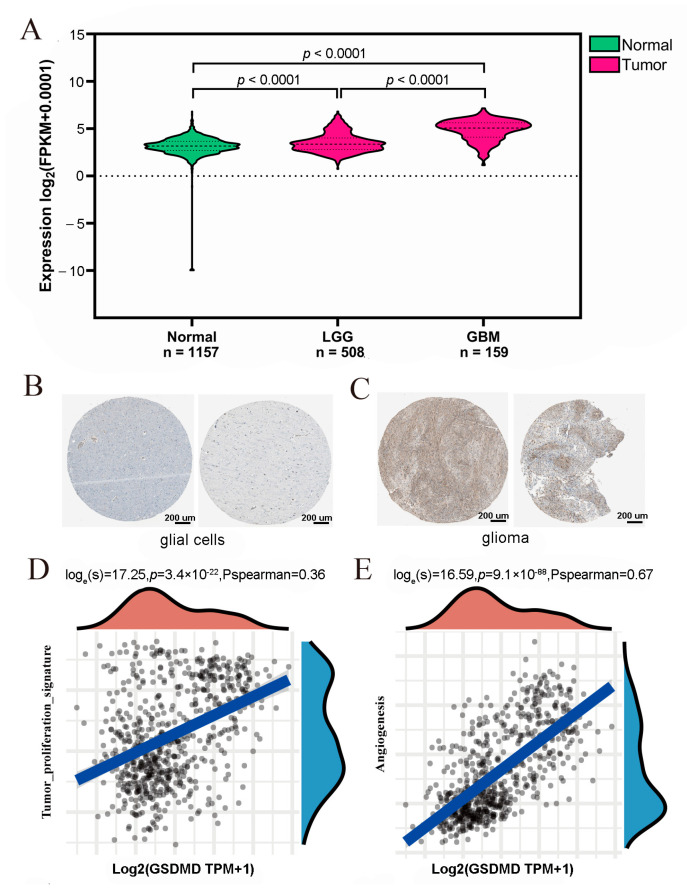
Gasdermin D (GSDMD) is significantly highly expressed in glioma tissues. (**A**) GSDMD is highly expressed in glioma tissues (LGG: low-grade glioma; GBM: glioblastoma/high-grade glioma) compared to the normal control. (**B**) The immunohistochemistry (IHC) results for the GSDMD antibody in normal glial tissues. (**C**) The IHC results for the GSDMD antibody in glioma tissues. (**D**,**E**) The association between proliferation and angiogenesis pathway scores and GSDMD expression levels was analyzed by Spearman correlation.

Based on the WHO classification, gliomas are divided into LGG and GBM [2,24]. In this study, we first analyzed the expression levels of GSDMD mRNA in 1157 normal brain tissues, 159 GBM tissues, and 508 LGG tissues. As shown in Figure 1A, the expression levels of GSDMD are significantly upregulated in the glioma tissues compared to the normal control group (*p* < 0.0001). Then, we also compared GSDMD at a protein level in normal glial cells and glioma cells using IHC. The results revealed that GSDMD was not detected in the normal glial cells, but glioma tissues were detected with medium or low staining (Figure 1B,C). In addition, it is well known that methylation is an important regulatory factor in gene expression [25]. Then, we further analyzed the GSDMD methylation in GBM primary tumors and normal tissues from the TCGA database, while data for LGG are not available. The results A) showed that the GSDMD methylation profile in glioma was lower than that of the control subjects (*p* = 0.215). In addition, after analyzing the signaling pathways using an R software GSVA package, the association between GSDMD expression levels and pathway scores were estimated by Spearman correlation. We also found that the activity of tumor proliferation signature and angiogenesis pathways was upregulated with the increase in GSDMD expression (*p* < 0.0001, Figure 1D,E).

These results demonstrated that GSDMD was highly expressed in glioma tissues both at mRNA and protein levels, and upregulated GSDMD promotes glioma development and progression.

### 3.2. High Levels of GSDMD Predict Poor Prognosis in Glioma Patients

**Figure 2:**
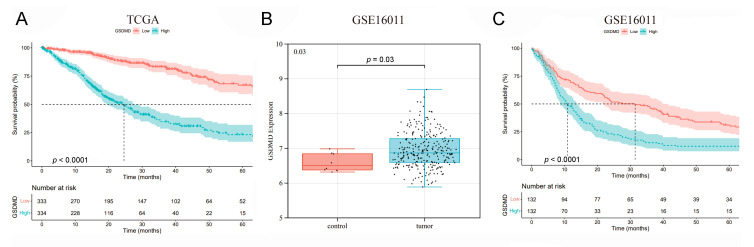
The prognostic value of gasdermin D (GSDMD) in glioma patients. (**A**) Highly expressed GSDMD indicated shorter overall survival in patients with glioma. (**B**) The high expression levels in cancerous tissues and (**C**) the prognostic role of GSDMD in glioma were verified in an independent GEO cohort (GSE16011).

**Figure 3:**
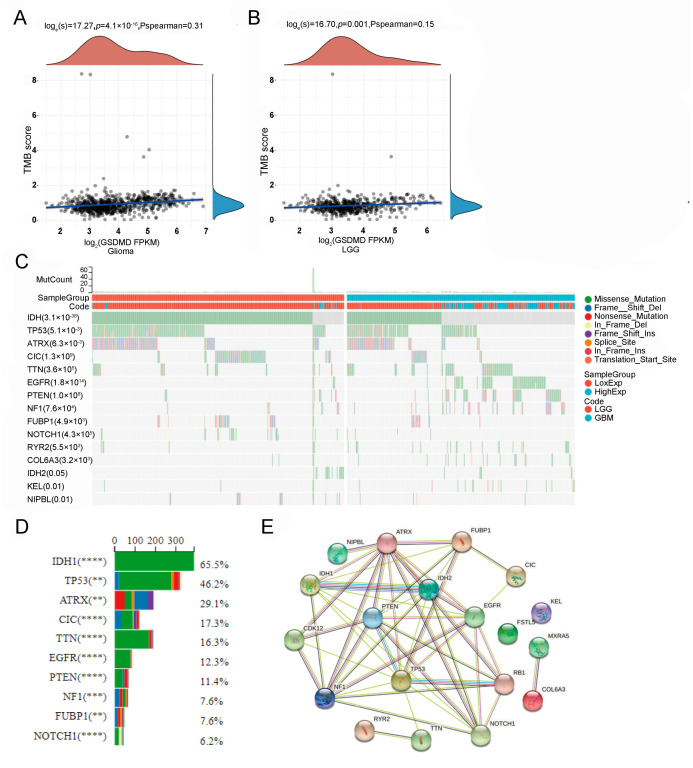
The tumor mutation burden (TMB) and genetic alteration analyses based on gasdermin D (GSDMD) expression in glioma patients. (**A**,**B**) A significantly positive correlation between GSDMD expression and TMB scores based on ‘ssgsea’ analysis was observed in patients with glioma and low-grade glioma (LGG) but not in glioblastoma (GBM). (**C**) The detailed information of the top 15 altered genes and variant classification associated with GSDMD expression. (**D**) The ratio of the top 10 altered genes that differ in the GSDMD low- and high-expression groups. (**E**) The protein–protein interaction (PPI) network of GSDMD-expression-associated altered genes. ** *p* < 0.01, *** *p* < 0.001, **** *p* < 0.0001.

To further investigate the relationship between GSDMD expression and clinical characteristics, we divided the cancer cases into GSDMD high- and low-expression according to the median expression of GSDMD and assessed the correlation of GSDMD expression with the OS of glioma patients. As shown in Figure 2A, high levels of GSDMD expression are linked to shorter OS for patients with glioma (*p* < 0.0001). In addition, we found patients with GSDMD mutation had a longer OS compared to nonmutation (*p* = 0.012, Supplementary Figure S1B). To further verify the cancer-promoting effects of GSDMD on glioma, we evaluated GSDMD levels and correlation with prognosis in patients with glioma in another cohort from GEO (GSE16011). Consistent with the results in the TCGA cohort, GSDMD had a higher expression level in glioma tissues compared to the normal control (*p* = 0.030, Figure 2B), and patients with high levels of GSDMD had shorter OS (*p* < 0.0001, Figure 2C) in the GSE16011 cohort. Subsequently, a multivariate Cox regression of gender, age, and GSDMD expression was performed. As shown in Supplementary Figure S2, GSDMD expression and age are independent prognostic factors for glioma patients (*p* < 0.0001, Figure 3). The above findings suggest that GSDMD expression is associated with OS in cancer cases with glioma.

### 3.3. GSDMD-Expression-Related Genetic Alteration

Human tumorigenesis is usually caused by the accumulation of genetic mutations. Then, we evaluated GSDMD levels and correlation with TMB scores using the R package GSVA with the method “ssgsea”. A total of 655 samples were detected with genetic alterations. As shown in Figure 3A–C, GSDMD expression had a positive association with TMB scores in patients with glioma (*p* < 0.0001, Figure 3A) and LGG (*p* = 0.001, Figure 3B). Then, the genetic alteration was compared in the GSDMD low- and high-expression groups and 31 genes with differential alteration were identified (Supplementary Table S1). The top 10 mutated genes included IDH1 (60.6%), TP53 (45.0%), ATRX (30.7%), CIC (16.9%), TTN (15.3%), EGFR (12.1%), PTEN (11.1%), NF1 (7.2%), FUBP1 (7.0%), and NOTCH1 (5.5%) (Figure 3C,D). The most frequent variant classification, variant type, and single nucleotide variant (SNV) class were a missense mutation (Supplementary Figure S3A), single nucleotide polymorphism (SNP, Supplementary Figure S3B), and C > T (Supplementary Figure S3C), respectively. In addition, the protein–protein interaction (PPI) network analysis was performed using the String database (https://cn.stringdb.org/, accessed on 5 March 2022) and we found that IDH1, PTEN, EGFR, and TP53 had widespread protein interactions, and other GSDMD-related altered genes also had extensive connections (Figure 3E).

**Figure 4:**
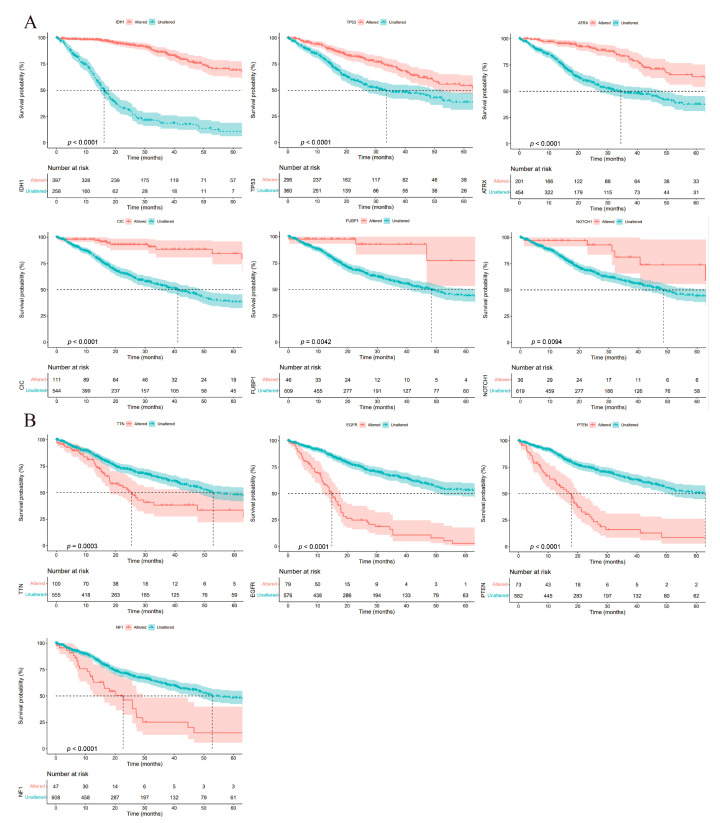
The prognostic value of gasdermin D (GSDMD)-related genetic alteration. (**A**) Six altered genes (IDH1, TP53, ARTX, CIC, FUBP1, and NOTCH1) negatively associated with GSDMD expression had a longer OS in patients with a genetic alteration. (**B**) Four genes (TTN, EGFR, PTEN, and NF1) positively associated with GSDMD expression had a longer OS in patients without genetic alteration.

Furthermore, the correlation between the top 10 altered genes and the patients’ OS was also analyzed. We found that six genes (IDH1, TP53, ARTX, CIC, FUBP1, and NOTCH1) had a higher alteration rate in the GSDMD low-expression group. Prognostic analysis revealed that patients with genetic alteration of the above six genes had a longer OS compared to the unaltered group (*p* < 0.0001: IDH1, TP53, ARTX and CIC; *p* = 0.0042: FUBP1; *p* = 0.0094: NOTCH1; Figure 4A). However, the other four genes (TTN, EGFR, PTEN, and NF1) had a higher alteration rate in the GSDMD high-expression group and patients with genetic alterations had a shorter OS (*p* < 0.0001: TTN, EGFR, PTEN and NF1; Figure 4B). These results are consistent with the finding that patients with high levels of GSDMD have a poor prognosis.

### 3.4. GSDMD Modulates Glioma Progression by Regulating the Immune Microenvironment

**Figure 5:**
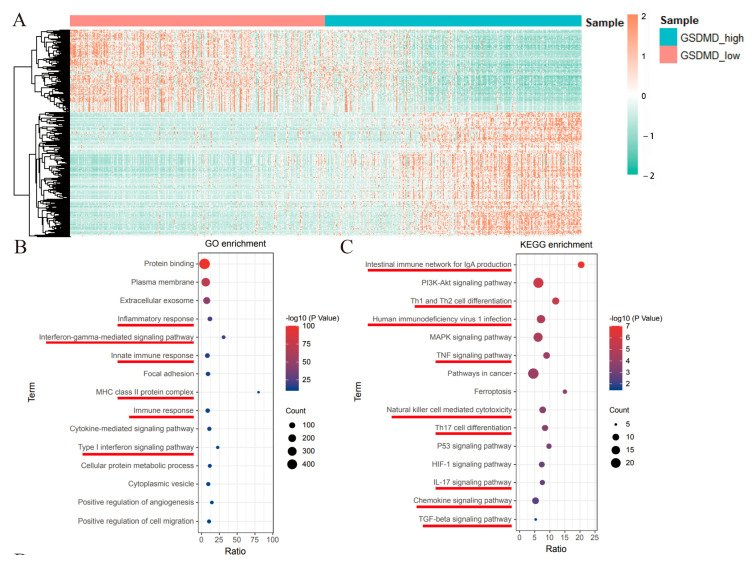
The differentially expressed genes (DEGs) between gasdermin D (GSDMD) the low- and high-expression groups and the GO/KEGG functional analyses. (**A**) The DEGs between the GSDMD low- and high-expression groups. The (**B**) GO and (**C**) KEGG enrichment analyses of these DEGs.

Alterations in the transcriptome also play a key role in the initiation and progression of tumors. Then, the DEGs between the GSDMD low-expression and high-expression groups were analyzed using the R package limma. A total of 731 DEGs (fold change >2.0 or <0.5; adj. *p*-value < 0.01) were identified (Figure 5A). To understand the functions of these DEGs, GO and KEGG functional enrichment analyses were performed using the Kobas database (http://kobas.cbi.pku.edu.cn/, accessed on 5 March 2022). The GO/KEGG enrichment analyses revealed that these DEGs were enriched to many immune regulatory pathways, such as inflammatory response, innate immune response, Th1 and Th2 cell differentiation, and tumor necrosis factor (TNF) signaling pathways (Figure 5B,C). In addition, GSEA analysis was also performed in the GSDMD low- and high-expression samples using GSEA software, and 20 gene sets were significantly enriched in GSDMD high-expression patients at a normal *p*-value < 0.01 (Supplementary Table S2). Of these, six immune-related gene sets were identified, including primary immunodeficiency, leukocyte transendothelial migration, and the intestinal immune network for IGA production (Supplementary Figure S4). These findings indicate that GSDMD exerts oncogenic effects on glioma and may do so via regulating the tumor immune microenvironment.

### 3.5. GSDMD Induces Pro-Tumor Immune Cell Filtration

**Figure 6:**
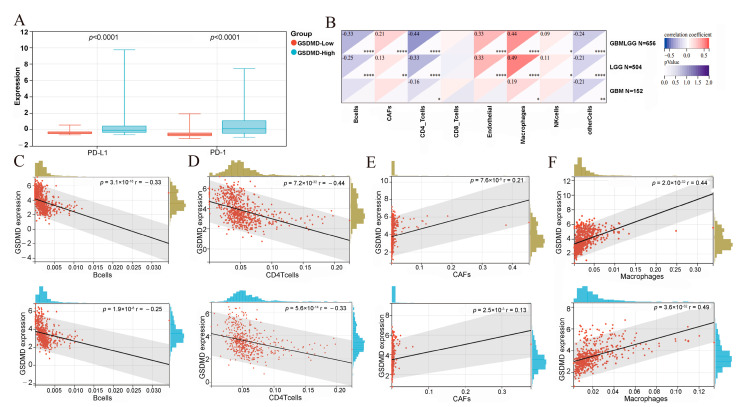
Immune infiltration analyses. (**A**) The expression levels of PD-1 and PD-L1 in gasdermin D (GSDMD) in the low- and high-expression groups. (**B**) The correlation of immune cell infiltration and GSDMD expression. (**C**,**D**) The ratio of B cells and CD4+ T cells decreased with increasing GSDMD expression. (**E**,**F**) Cancer-associated fibroblasts and macrophages increased with increasing GSDMD expression. * *p* < 0.05, ** *p* < 0.01, **** *p* < 0.0001.

Considering the role of GSDMD in the modulation of immune-related signaling pathways, we analyzed the relationship between GSDMD and the tumor immune microenvironment. Recently, PD-1 and PD-L1 cascade have been well studied and considered as important representatives of the tumor immune microenvironment [26,27]. Then, we first analyzed the expression levels of PD-1 and PD-L1 in the GSDMD low- and high-level glioma patients and found that both PD-1 and PD-L1 had significant upregulation in the highly expressed GSDMD patients (*p* < 0.0001, Figure 6A). Subsequently, we applied EPIC algorithms to evaluate the correlation between the GSDMD expression and infiltration levels of different immune cells. As shown in Figure 6B, there was a significant difference in immune cell infiltration in the GSDMD low- and high-expression patients. In patients with glioma, highly expressed GSDMD was negatively correlated with B cell (*p* < 0.0001, Figure 6C) and CD4+ T cell (*p* < 0.0001, Figure 6D) infiltrations and positively correlated with cancer-associated fibroblasts (CAFs) (*p* < 0.0001, Figure 6E) and macrophages (*p* < 0.0001, Figure 6F). Because B cells [28] and CD4+ T cells [29] usually inhibit tumor growth while CAFs [30] and macrophages [30] promote tumor growth, it was concluded that high levels of GSDMD induced an immunosuppressive microenvironment and promoted glioma progression.

### 3.6. Single-Cell Analysis

**Figure 7:**
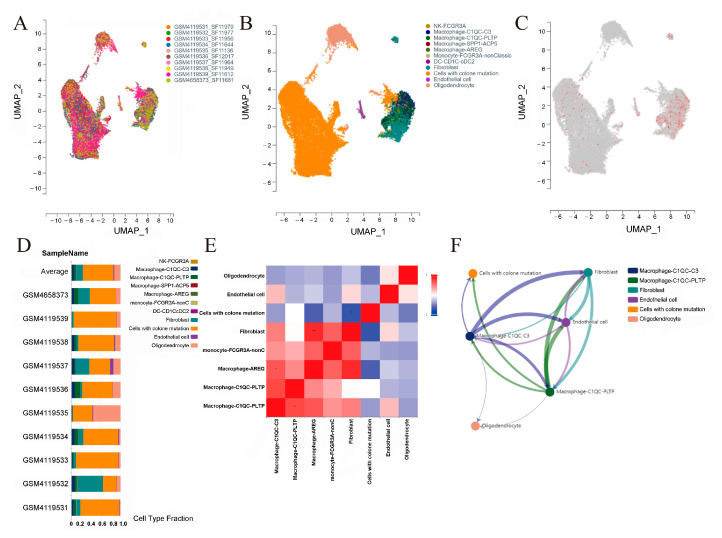
Single-cell analyses. (**A**) UMAP plot showing the single-cell profile based on the glioma samples. (**B**) UMAP plot showing the cell lineages in glioma. (**C**) UMAP plot showing the gasdermin D (GSDMD) expression for cell lineages in glioma. (**D**) The ratio of identified cells in the glioma samples. (**E**) Pearson correlation analyses of these identified cells in glioma. (**F**) Intercellular communication among the identified cells in glioma with more than 35 interaction strengths.

To further explore the correlation among GSDMD, CAFs, and macrophages in glioma, we performed a single-cell analysis on 10 untreated glioma samples (Figure 7A). A total of eight cell types (Figure 7B), including macrophages, monocytes, natural killer (NK) cells, CAFs, endothelial cells, dendritic cells (DC), cells with colon mutation, and oligodendrocytes, were identified. Then, we analyzed GSDMD expression based on cell clusters and found that GSDMD was mainly expressed in the CAF and macrophage clusters (Figure 7C), which may be the potential molecular mechanism for increased CAFs and macrophages in the GSDMD high-expression group. Then, we calculated the cell type composition of each sample and revealed that CAFs and macrophages were the dominant immune components with approximate 14% and 9% average ratios (Figure 7D), respectively. Furthermore, the Pearson correlation analysis revealed that CAFs and AREG-type macrophages showed significantly positive correlations (*p* < 0.01, Figure 7E). In addition, the results of cell communication also confirmed the connection between CAFs and macrophages (Figure 7F). Therefore, the single-cell analysis indicated that GSDMD promotes glioma progression by regulating CAFs and macrophages.

### 3.7. GSDMD Knockdown Inhibits the Proliferation and Migration of Glioma Cells

**Figure 8:**
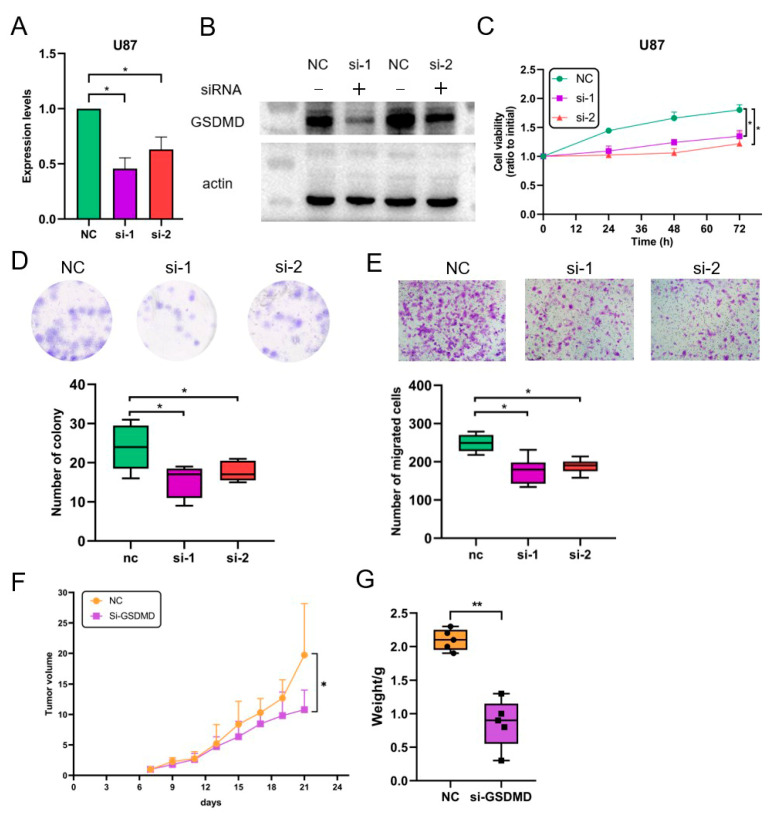
In vitro and in vivo experiments revealed that gasdermin D (GSDMD) knockdown inhibited glioma progression. (**A**,**B**) Lower expression levels of GSDMD mRNA and protein were detected in glioma cells transfected with GSDMD siRNAs. (**C**) The CCK-8 assays found GSDMD knockdown reduced cell viability in glioma. (**D**) Colony formation assays revealed that knocking down GSDMD decreased the number of formed clones in the glioma cells. (**E**) The transwell assay results found that fewer glioma cells transfected with GSDMD-siRNAs migrated from the transwell membrane than in the NC group. (**F**,**G**) The xenograft tumorigenicity experiment revealed that GSDMD knockdown suppressed tumor growth in vivo. * *p* < 0.05, ** *p* < 0.01.

To further verify the role of GSDMD in glioma, two GSDMD knockdown glioma cell lines were constructed. Then, the expression levels of GSDMD were detected and the results showed significantly lower expression in U87 and A172 glioma cells treated with si-GSDMD (Figure 8A, Supplementary Figure S5A), which was also confirmed by the WB assay (Figure 8B). In addition, CCK-8, colony formation, and transwell assays were performed to investigate the impact of GSDMD on glioma cells. The CCK-8 results showed that GSDMD knockdown inhibited the proliferation (Figure 8C, Supplementary Figure S5B) of glioma cells. The colony formation assays showed GSDMD knockdown decrease the number of cell clones (Figure 8D). In addition, the transwell assays revealed that knocking down GSDMD inhibited the migration (Figure 8E, Supplementary Figure S5C) of glioma cells.

To further verify the effects of GSDMD on glioma, we used NC and si-GSDMD tumor-bearing nude mice, and five mice were used in each group. After 21 days of tumor transplant, the mice were sacrificed to analyze the volume and weight of their tumor nodules. The tumor volume was significantly smaller in the GSDMD knockdown group than that in the NC group (Figure 8F, Supplementary Figure S5D). Furthermore, a lower weight was also detected in the GSDMD knockdown group (Figure 8G). K-M survival analysis showed that GSDMD knockdown tumor-bearing mice had prolonged survival (Supplementary Figure S5E).

In addition, previous studies have revealed cell subtypes that are associated with glioma progression [31,32], and EGFR, NF1, and PDGFRA/IDH1 are markers for the classical, mesenchymal, and proneural types, respectively. Then, we analyzed the correlation of expression levels between GSDMD and these genes using linear regression analysis. The result showed GSDMD levels were positively associated with EGFR, the marker gene of the classical type (Supplementary Figure S6). Therefore, GSDMD also plays a role in regulating the cell states of glioma. These results confirmed the oncogenic role of GSDMD in glioma and revealed that inhibiting GSDMD may be an effective strategy in the treatment of glioma.

### 3.8. Competing Endogenous RNA (ceRNA) Network Analysis

**Figure 9:**
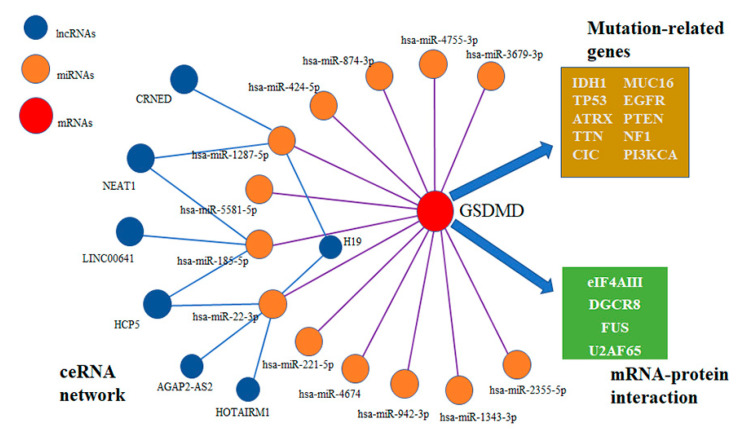
Competing endogenous RNA (ceRNA) network of gasdermin D (GSDMD), binding microRNAs, and long non-coding RNAs. Moreover, GSDMD binding proteins and related genetic alterations are also shown.

RNA post-transcriptional modification plays an important role in gene function and the ceRNA regulatory network has been well understood over the past few years. We first analyzed the differentially expressed miRNAs between the GSDMD low- and high-level samples from the TCGA database using R package limma, and 351 miRNAs were identified (** *p* < 0.01). Then, miRNAs with binding sites with GSDMD were identified using the TargetScan v7.2 database (http://www.targetscan.org/vert_72/, accessed on 5 March 2022), and the intersection with the above 351 miRNAs was screened (Supplementary Figure S6A). As shown in Supplementary Figure S6B, 13 miRNAs with GSDMD targets were selected. Similarly, we also identified the differentially expressed long non-coding RNAs (lncRNAs) and integrated the lncRNA–miRNA regulatory interactions using the lncBase database (https://diana.e-ce.uth.gr/lncbasev3, accessed on 5 March 2022). In addition, proteins that interacted with the GSDMD mRNA were also predicted by the Starbase v2.0 database (https://starbase.sysu.edu.cn/starbase2, accessed on 5 March 2022). Finally, we summarized the network of molecules that interacted with GSDMD in Figure 9, including the ceRNA network, GSDMD mRNA-binding proteins (RBP), and GSDMD-related genetic alterations.

## 4. Discussion

Previous studies have considered surgical resection with postoperative chemotherapy and radiotherapy as a standard treatment modality for patients with glioma [33]. However, glioma patients benefit little from standard therapy [34]. Therefore, there is an urgent need to understand the molecular mechanisms of glioma genesis and search for more efficacious biomarkers and treatment strategies for glioma diseases. In this study, we found that the expression of GSDMD in glioma tissues is significantly higher than that in the corresponding control tissues both at mRNA and protein levels. Moreover, the glioma patients with high expression of GSDMD had shorter OS. In addition, we explored the modulatory mechanisms of GSDMD in glioma progression. We found that GSDMD expression is related to genetic mutations in glioma, and highly expressed GSDMD indicated a pro-tumor immune microenvironment with more infiltrated CAFs and macrophages. These results suggest that GSDMD is a potential prognosis biomarker and therapeutic target for glioma patients.

GSDMD is a precursor of a pore-forming protein that exerts important effects on host defense against danger signals and pathogen infection [7,35]. The N-terminal moiety of GSDMD can bind to membranes and form pores, triggering pyroptosis [5]. However, the specific role of GSDMD in cancer initiation and development is still not clear. Gao and coworkers found that GSDMD is highly expressed in non-small cell lung cancer compared to matched adjacent tumor specimens and is associated with a poor prognosis, whereas low-level expression of GSDMD in gastric cancer promotes tumor proliferation and occurrence [36]. Moreover, GSDMD activation can exert anti-tumor effects on endometrial cancer [37]. Thus, the precise roles and mechanisms of GSDMD in tumorigenesis remain elusive [38,39].

In this study, we found that GSDMD played an oncogenic role in glioma and can be used as an independent prognostic factor. A previous study confirmed that GSDMD is highly expressed in non-small cell lung cancer (NSCLC), and the expression level of GSDMD was associated with tumor size and stage [10]. Knockdown of GSDMD suppressed tumor proliferation by promoting the mitochondrial apoptotic pathway and inhibiting the EGFR/Akt signaling pathway in NSCLC. Therefore, targeting GSDMD may be a novel approach to the treatment of cancer. In this study, we constructed GSDMD knockdown U87 and A172 glioma cells, and the functional experiment results showed GSDMD knockdown inhibited the proliferation and migration of glioma cells in vitro. Furthermore, we conducted experiments with nude mice bearing NC and si-GSDMD U87 glioma cells, and further studies showed GSDMD knockdown suppressed tumor growth in vivo. Therefore, GSDMD knockdown could inhibit glioma progression both in vitro and in vivo.

Previous studies have found that TMB, a genetic alteration at the whole genome level, is related to tumor progression and the prognosis of patients with glioma [40,41]. In glioma, high levels of TMB indicate a poor prognosis [40]. Then, we analyzed the correlation between TMB and GSDMD expression, and the results exhibited a significantly positive association. Furthermore, we also found that patients with GSDMD expression with positively associated genetic alterations had a shorter OS, while negatively associated genetic alterations indicated a longer OS. These results suggested that highly expressed GSDMD could regulate genetic alterations and increase TMB, thereby causing poor prognosis in glioma patients.

Recently, targeting TME has attracted researchers’ interest in the treatment of tumors, as well as the immune checkpoints [42]. The immune system exerts important effects on tumor development and progression [43]. Immune cells can either promote tumor growth and metastasis or inhibit tumor growth and facilitate its destruction [44,45,46]. In addition, the balance between these two opposing roles is determined by a complex interplay between different immune cells and molecules. Pro-tumor immune cells are typically cells that have been co-opted by the tumor to promote its survival and growth, such as regulatory T cells, myeloid-derived suppressor cells, and TAMs [46]. Anti-tumor immune cells are cells that can recognize and attack cancer cells, such as CD8+ T cells and NK cells [13,47]. The balance between pro-tumor and anti-tumor responses is also regulated by the release of pro-inflammatory and anti-inflammatory molecules [48].

Moreover, the recent development of multi-color flow cytometry and single-cell sequencing technologies have enabled the analysis of tumor microenvironmental components at a higher resolution [12,13]. Moreover, bioinformatic algorithms for analyzing immune components in tumor tissues have also been developed, such as EPIC [49]. In the present study, the functional analyses of DEGs in GSDMD low- and high-expression patients found that many immune-related pathways were enriched. Thus, we speculated that GSDMD regulated glioma progression by influencing the tumor immune microenvironment. Furthermore, GSEA analysis confirmed the immunomodulatory effects of GSDMD on glioma progression. Then, we performed EPIC analyses to characterize the immune composition in GSDMD low- and high-expression glioma. The results showed that highly expressed GSDMD induces an immunosuppressive microenvironment by increasing the ratio of CAFs and macrophages but reducing CD4+ T cells and B cells. Furthermore, the results of single-cell data in 10 glioma tissues confirmed the positive association among GSDMD expression, CAFs, and macrophages. CAFs and macrophages in the TME of glioma also had a positive regulatory correlation. Therefore, we summarized that GSDMD can promote glioma progression by increasing the ratio of CAFs and macrophages to induce an immunosuppressive TME.

## 5. Conclusions

GSDMD was more highly expressed in glioma tissues compared to normal tissues, and a higher level of GSDMD indicated a shorter OS in patients with glioma. Moreover, GSDMD expression was significantly associated with tumor mutation burden, genetic alterations, and immune cell infiltration. Highly expressed GSDMD promoted a permissive microenvironment for tumor growth by upregulating the ratio of CAFs and macrophages in the TME. GSDMD knock-down inhibited the progression of glioma both in vitro and in vivo. Therefore, GSDMD is a promising prognostic biomarker and a novel therapeutic target for patients with glioma.

## Data Availability

The original data presented in this study are included in the article or Supplementary Materials; for other information, requests can be directed to the corresponding authors.

## Supplementary Materials

The following supporting information can be downloaded at: https://www.mdpi.com/article/10.3390/biom13060904/s1, Figure S1: (A) The promoter methylation level of GSDMD in glioblastoma (GBM). (B) Glioma patients with GSDMD mutation had longer overall survival. Figure S2: The forest plot showed the results of the multivariate Cox regression of age, gender, and GSDMD expression in glioma patients. Figure S3: The most frequent variant classification, variant type, and single nucleotide variant (SNV) class were a missense mutation (A), single nucleotide polymorphism (B), and C > T (C), respectively. Figure S4: Gene set enrichment analyses (GSEA). Six immune-related gene sets were enriched in the GSDMD high-expression group. Figure S5: (A) Lower expression levels of GSDMD mRNA were detected in glioma cells transfected with GSDMD siRNAs. (B) The CCK-8 assays found GSDMD knockdown reduced cell viability. (C) The transwell assay results found that fewer glioma cells transfected with GSDMD-siRNAs migrated from the transwell membrane than in the NC group. (D) The tumor volume was significantly smaller in the GSDMD knockdown group than that in the NC group. (E) The K-M survival analysis showed that GSDMD knockdown tumor-bearing mice had prolonged survival. Figure S6: The correlation of expression levels between GSDMD and EGFR, NF1, PDGFRA, and IDH1 using linear regression analysis. Figure S7: (A) The intersection between differentially expressed miRNAs in the GSDMD low- and high-expression groups and binding miRNAs predicted by the TargetScan v7.2 database. (B) The heatmap of the 13 screened miRNAs targeting GSDMD. Table S1: 31 genes with differential alteration were identified in the GSDMD low- and high-expression groups, Table S2: 20 gene sets were significantly enriched in GSDMD high-expression patients.
