# Supplementary material for: Gasdermin D Plays an Oncogenic Role in Glioma and Correlates to an Immunosuppressive Microenvironment
Source: Biomolecules. 2023 May 29;13(6):904. doi: 10.3390/biom13060904 (PMC10295969; doi:10.3390/biom13060904)
Supplement: Supplementary file 1 [file biomolecules-13-00904-s001.zip › biomolecules-2321548-supplementary.pdf]

Supplementary Materials:

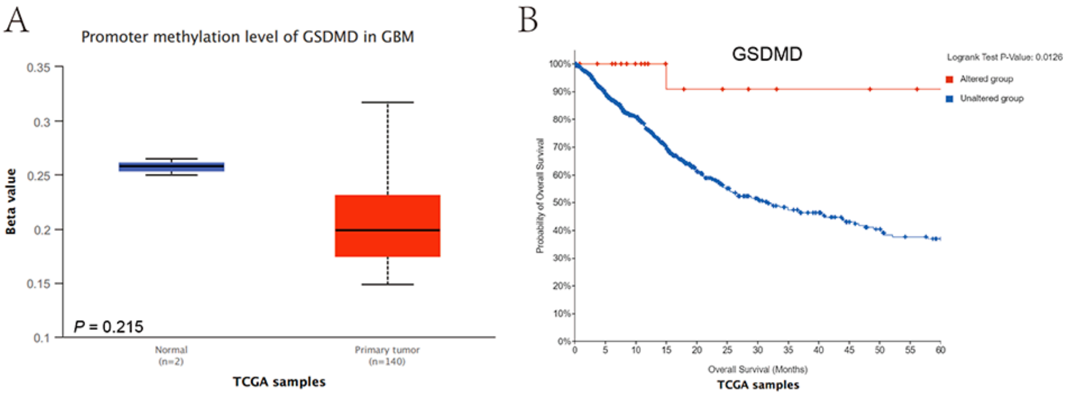

Figure S1: (A) The promoter methylation level of GSDMD in glioblastoma (GBM). (B) Glioma patients with GSDMD mutation had longer overall survival.

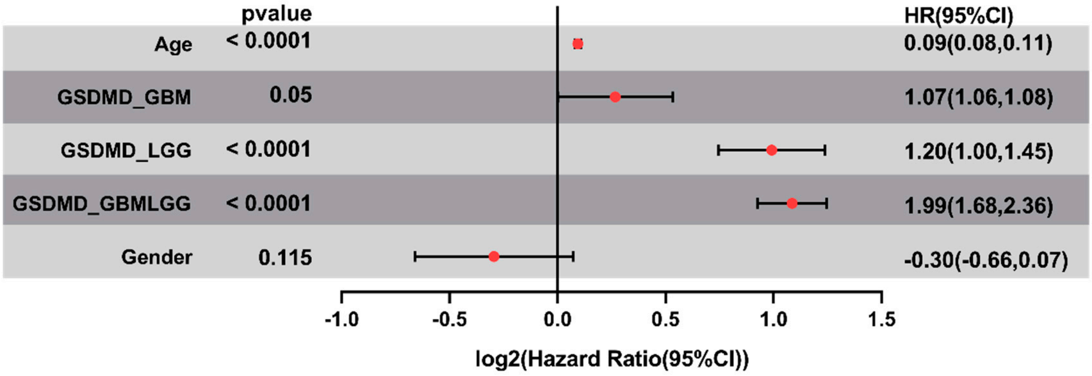

Figure S2: The forest plot showed the results of the multivariate Cox regression of age, gender, and GSDMD expression in glioma patients.

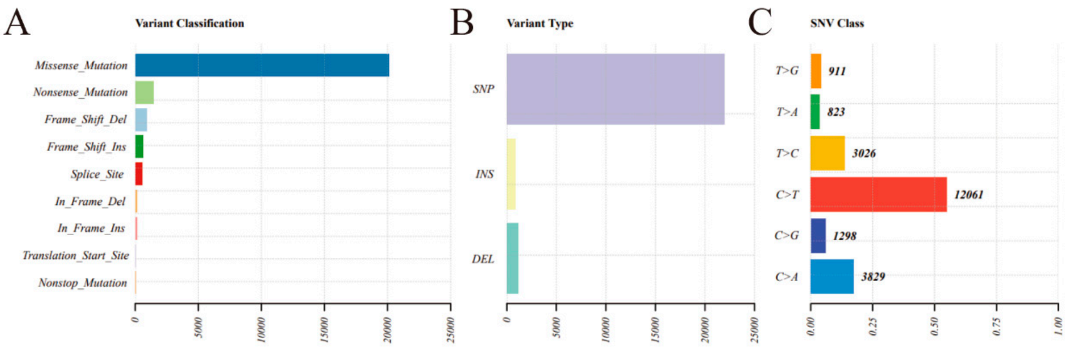

Figure S3: The most frequent variant classification, variant type, and single nucleotide variant (SNV) class were a missense mutation (A), single nucleotide polymorphism (B), and C>T (C), respectively.

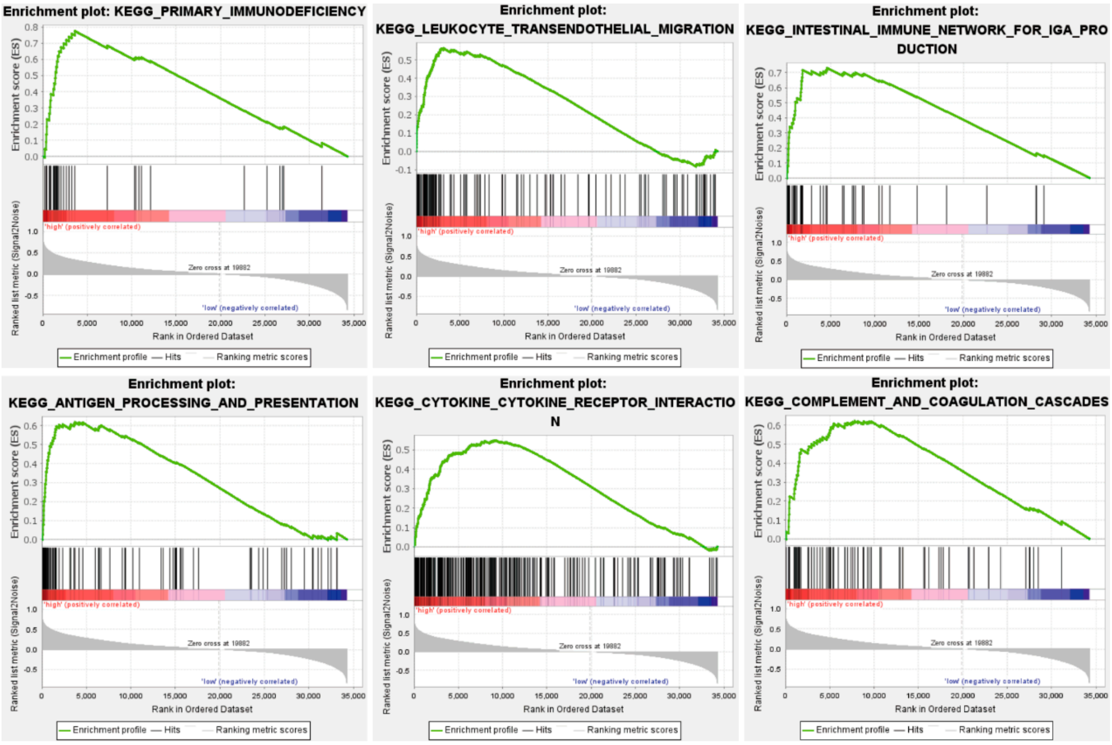

Figure S4: Gene set enrichment analyses (GSEA). Six immune-related gene sets were enriched in the GSDMD high-expression group.

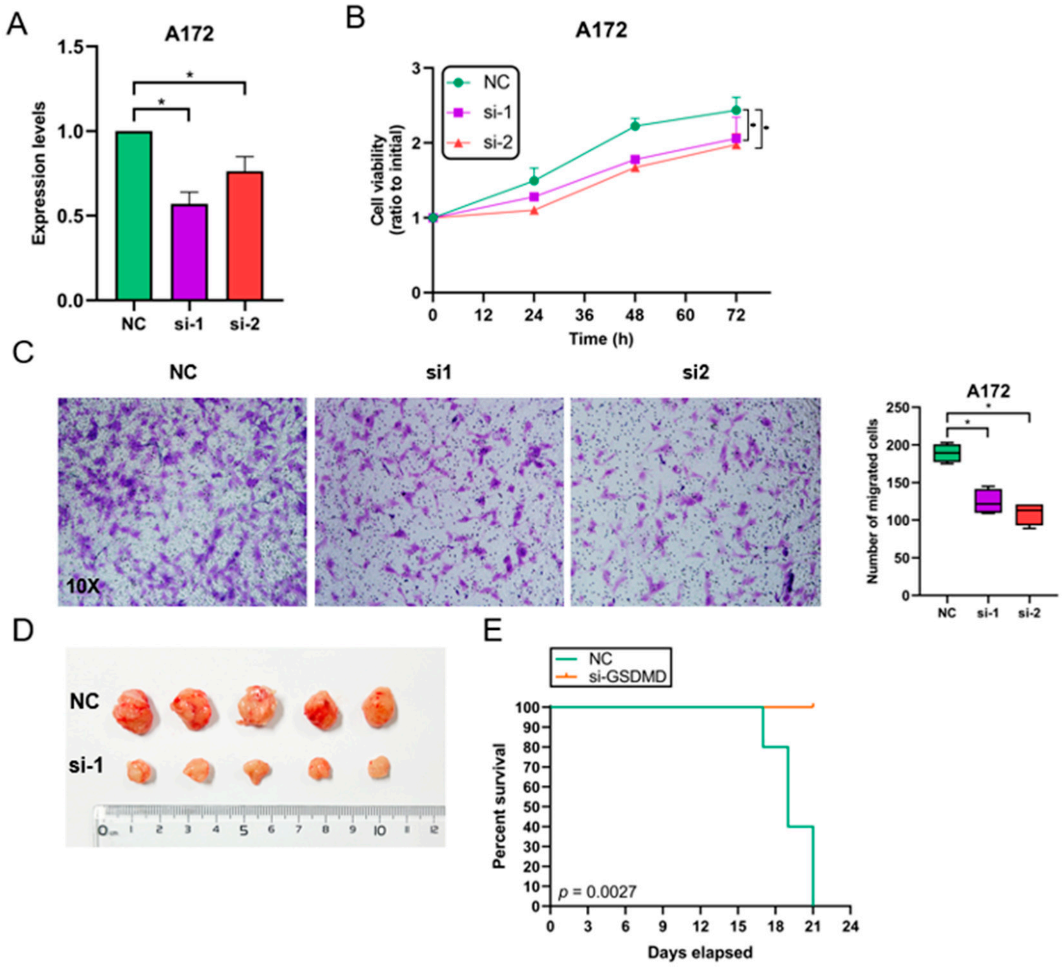

Figure S5: (A) Lower expression levels of GSDMD mRNA were detected in glioma cells transfected with GSDMD siRNAs. (B) The CCK-8 assays found GSDMD knockdown reduced cell viability. (C) The transwell assay results found that fewer glioma cells transfected with GSDMD-siRNAs migrated from the transwell membrane than in the NC group. (D) The tumor volume was significantly smaller in the GSDMD knockdown group than that in the NC group. (E) The K-M survival analysis showed that GSDMD knockdown tumor-bearing mice had prolonged survival. * $p < 0.05$.

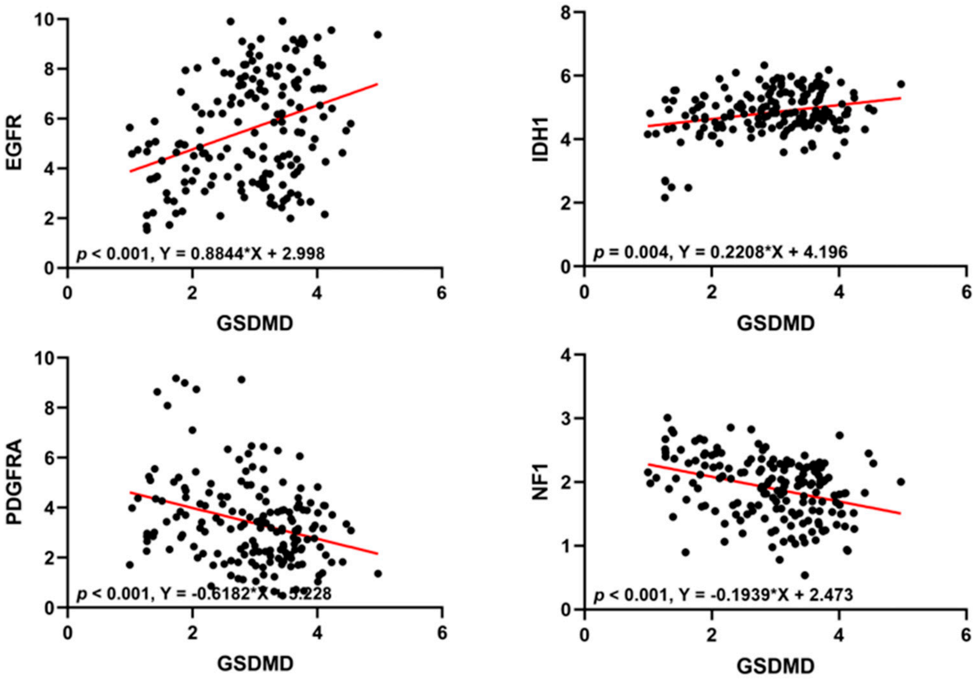

Figure S6: The correlation of expression levels between GSDMD and EGFR, NF1, PDGFRA, and IDH1 using linear regression analysis.

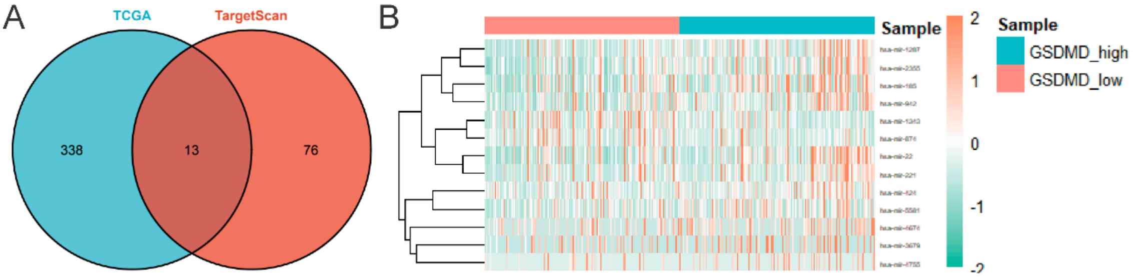

Figure S7: (A) The intersection between differentially expressed miRNAs in the GSDMD low- and high-expression groups and binding miRNAs predicted by the TargetScan v7.2 database. (B) The heatmap of the 13 screened miRNAs targeting GSDMD.

Gene	MutCount	*p*
IDH1	390	3.13E-36
TP53	275	0.005081
ATRX	173	0.006251
CIC	103	1.30E-09
TTN	97	3.56E-05
EGFR	73	1.85E-14
PTEN	68	1.02E-08
FUBP1	45	0.004899
NF1	45	0.000759
NOTCH1	37	4.27E-05
RYR2	30	0.005464
COL6A3	23	0.00318
IDH2	22	0.048352
KEL	20	0.013231
NIPBL	20	0.011752
RB1	20	0.013231
MXRA5	12	0.009153
SPATA31E1	10	0.024729
FSTL5	8	0.012299
CDK12	7	0.02188
NHS	7	0.02188
AK7	6	0.041411
ATP4A	6	0.039194
CSF2RA	6	0.039194
DOCK2	6	0.041411
PCDHAC1	6	0.039194
PRKG2	6	0.041411
SCN2A	6	0.039194
UMODL1	6	0.041411
UNC13A	6	0.039194
ZNF148	6	0.039194

Table S1: 31 genes with differential alteration were identified in the GSDMD low- and high-expression groups.

GS follow link to MSigDB	ES	NES	FDR q-val
PRIMARY_IMMUNODEFICIENCY	0.78	2.04	0.025
AUTOIMMUNE_THYROID_DISEASE	0.73	1.97	0.034
GLUTATHIONE_METABOLISM	0.63	1.95	0.03
HEMATOPOIETIC_CELL_LINEAGE	0.66	1.95	0.024
LEISHMANIA_INFECTION	0.69	1.94	0.02
LEUKOCYTE_TRANSENDOTHELIAL_MIGRATION	0.56	1.94	0.017
VIRAL_MYOCARDITIS	0.61	1.9	0.027
LYSOSOME	0.56	1.89	0.025
ANTIGEN_PROCESSING_AND_PRESENTATION	0.62	1.88	0.025
AMINO_SUGAR_AND_NUCLEOTIDE_SUGAR_METABOLISM	0.6	1.88	0.023
INTESTINAL_IMMUNE_NETWORK_FOR_IGA_PRODUCTION	0.73	1.84	0.033
ALLOGRAFT_REJECTION	0.79	1.82	0.04
APOPTOSIS	0.49	1.81	0.039
TOLL_LIKE_RECEPTOR_SIGNALING_PATHWAY	0.51	1.81	0.037
CYTOSOLIC_DNA_SENSING_PATHWAY	0.55	1.81	0.036
PROTEASOME	0.73	1.8	0.036
CYTOKINE_CYTOKINE_RECEPTOR_INTERACTION	0.55	1.79	0.039
COMPLEMENT_AND_COAGULATION_CASCADES	0.62	1.78	0.042
PANTOTHENATE_AND_COA_BIOSYNTHESIS	0.63	1.78	0.04
GALACTOSE_METABOLISM	0.58	1.73	0.047

Table S2: 20 gene sets were significantly enriched in GSDMD high-expression patients.
